# Interventions employing mobile technology for overweight and obesity: an early systematic review of randomized controlled trials

**DOI:** 10.1111/obr.12006

**Published:** 2012-11-20

**Authors:** R Bacigalupo, P Cudd, C Littlewood, P Bissell, M S Hawley, H Buckley Woods

**Affiliations:** School of Health and Related Research (ScHARR), University of SheffieldSheffield, UK

**Keywords:** mobile technology, obesity, overweight, weight loss

## Abstract

Obesity is a global epidemic with major healthcare implications and costs. Mobile technologies are potential interventions to promote weight loss. An early systematic review of this rapidly growing area of research was conducted. Electronic databases were searched for articles published between January 1998 and October 2011. Data sources included Medline, Embase and the Cochrane Central Register of Controlled Trials. Ongoing research was searched for using clinical trials databases and registers. Out of 174 articles retrieved, 21 met the inclusion criteria of randomized controlled trials (RCTs) on mobile technology interventions facilitating weight loss in overweight and obese adults with any other comparator. A narrative synthesis was undertaken. Seven articles were included and appraised using the Cochrane risk of bias tool: four presented a low risk of bias and three presented a high risk of bias. There is consistent strong evidence across the included multiple high-quality RCTs that weight loss occurs in the short-term because of mobile technology interventions, with moderate evidence for the medium-term. Recommendations for improving the reporting and quality of future trials are made including reporting weight loss in percent to meet clinical standards, and including features such as long-term follow-up, cost-effectiveness and patient acceptability.

## Introduction

Populations who are overweight or obese have a higher likelihood of serious health complications including type 2 diabetes, stroke, cardiovascular diseases, cancer and osteoarthritis 1–3. Obesity has increased in almost all countries worldwide 4 and is now considered to constitute a global epidemic 2. Three out of four Americans and seven out of 10 Britons could be overweight or obese by 2020 3. Almost 25% of adults in the United Kingdom are obese 5 and recent modelling estimates that over half of the UK adult population could be obese by 2050 costing the National Health Service (NHS) £10 billion per year 6. Yearly, extra healthcare costs of obesity in the USA were 75 billion dollars in 2003, and approximately 33 billion euros per year in the European Union in 2002 3. Clinically significant weight loss is seen as a priority, and a reduction in an individual's weight of 5–10% is associated with an improvement in the clinical risk of adverse health problems 7–9.

Currently, commissioned weight loss services and interventions in Europe and the USA often start by considering the use of an appropriate combination of diet, exercise programme and counselling for the individual – drawing on behaviour change theories 10,11. There is increasing interest in the use of digital technologies to support individuals in their healthcare decisions 12,13 because interactive technology can be designed and used to persuade people to modify their attitudes or behaviours and to enhance levels of surveillance over behaviours 14,15. Additionally, information and communication technologies have the potential to provide acceptable and cost-effective interventions by transferring treatment, rehabilitation and prevention of obesity to self-care in the community 16–18. In general, self-care in the community, especially when employing technology, frequently involves self-monitoring; which fits well as self-monitoring has been described as the cornerstone of effective behavioural weight loss intervention programmes 19.

A systematic review focusing on self-monitoring activities in weight loss reported on the use of digital technology in five out of 22 studies, the five included use of the Internet, personal digital assistants (PDAs; handheld computers) and electronic digital scales 19. The case for employing mobile electronic technologies has been stated to be that it has the advantage of portability and can be used outside the healthcare setting (and the home) as people go about their daily lives 13,20. The reason for carrying out this systematic review is that there are none to date that focus exclusively on mobile technologies for weight loss. However, there are published systematic reviews of mobile technology being employed in interventions across various health conditions such as smoking cessation, weight loss, anxiety, diabetes management, eating disorders, alcohol use, and healthy eating and physical activity. The results of these reviews are varied. One review 20 found limited short-term evidence for weight loss from four studies published between 1985 and 2009. Another review 21 studied healthcare provision and disease management support via mobile phone and text messaging interventions. The latter review covered 12 clinical areas (such as smoking cessation, physical activity, asthma and diabetes), conducted in 13 countries, and found improved health outcomes in key areas such as improved asthma symptoms, medicine compliance and health education, but a need for larger and more robust controlled trials 21. In a similar vein a protocol was recently published that promises a systematic review of the effectiveness of mobile technology in a broad range of worldwide health care and public health 12.

While these reviews may include data directly relevant to weight loss interventions, their main goal was to draw inferences from results across health conditions and other outcomes, therefore providing a more limited focus on weight loss. Wide-ranging technology-based weight loss interventions (e.g. cell phones/Internet) have been reviewed 10 and encompassed research methodologies other than randomized controlled trials (RCTs), but did not discuss at all the quality of evidence or consider details of good trial design, e.g. factors such as risk of bias. From the 24 studies considered in the review 10, five key components for effective technology-based weight loss interventions were identified: self-monitoring, counsellor feedback and communication, social support, use of a structured programme, and use of an individually tailored programme. The key components were selected by Khaylis *et al*. 10 from 21 study findings because they indicated the most consistent association with successful weight loss interventions. The mobile technologies such as portable body monitors, pedometers and handheld PDAs combined with self-monitoring were associated with weight loss, thus providing evidence of the benefits of mobile technology 10.

Use of digital mobile technologies in healthcare while increasingly common is still a relatively new modality, and relatively few RCTs have been published exploring their effectiveness. There is therefore a need for a review of interventions employing mobile digital technologies. Such reviews need to consider the strength of the evidence presented by published studies when informing their design and the evidence already published (if any) for the intervention they plan to investigate. Well-designed RCTs, especially double-blinded ones, are widely regarded as the best 22 whereas non-randomized and unblinded trials may significantly overestimate outcomes 23.

In the light of the published reviews and discussion mentioned earlier, the aim of this systematic review was not only to study in more detail whether mobile technology is an appropriate medium for facilitating weight loss in overweight and obese adults, but also to identify, compare and contrast the features of the studies and interventions to inform future RCT study design.

## Methods

The systematic review was conducted using an agreed, but unpublished, predetermined protocol and in accordance with the Preferred Reporting Items for Systematic Reviews and Meta-analysis (PRISMA) statement 24.

### Data sources and search strategy

A systematic search was undertaken to identify RCTs in the area of mobile technology and obese adults. Data sources were Medline, Embase, Science Citation Index, Social Science Citation Index, Cumulative Index to Nursing and Allied Health Literature, the Cochrane Central Register of Controlled Trials, Meta register of controlled trials and Clinical Trials.gov. Keywords and subject heading terms for mobile technology were combined with terms relating to obesity or weight management. The search was limited to RCTs for the period between 1998 and October 2011. There were no RCT search results on Medline before 1998 and given the resources available we decided to set this date limit on the search. The Medline search strategy is shown in Table 1. The electronic search was complemented by hand-searching the reference lists of the articles found and previous systematic reviews in order to identify other relevant evidence not found through electronic database searching.

**Table 1:** Medline example literature search strategy

Search	Keywords
1.	(Mobile technology or m-health or mhealth or personal digital assistants or PDA* or mobile phone* or hand-held computers or pocket computers or PalmPilots or assistive technology or telecare or telehealth or telemonitoring).ti,ab (9,536).
2.	telemedicine/ (7,995)
3.	1 or 2 (16,291)
4.	(obesity or weight management or BMI).ti,ab (124,779).
5.	weight loss/ or obesity/ (107,873)
6.	4 or 5 (171,008)
7.	3 and 6 (126)
8.	Clinical trial/ (460,598)
9.	Randomized controlled trial/ (301,753)
10.	Randomization/ (70,565)
11.	Single blind procedure/ (0)
12.	Double blind procedure/ (0)
13.	Crossover procedure/ (0)
14.	Placebo/ (0)
15.	Randomi?ed controlled trial$.tw (49,269).
16.	Rct.tw (4,856).
17.	Random allocation.tw (899).
18.	Randomly allocated.tw (12,902).
19.	Allocated randomly.tw (1,518).
20.	(allocated adj2 random).tw (673).
21.	Single blind$.tw (8,976).
22.	Double blind$.tw (98,254).
23.	([treble or triple] adj blind$).tw (219).
24.	Placebo$.tw (130,532).
25.	Prospective study/ (290,434)
26.	or/8–25 (879,782)
27.	Case study/ (1,506,736)
28.	Case report.tw (164,891).
29.	Abstract report/ or letter/ (723,199)
30.	or/27–29 (2,103,244)
31.	26 not 30 (857,094)
32.	7 and 31 (24)
33.	limit 32 to yr = ‘1998-Current’ (24)

### Study selection

The inclusion/exclusion criteria are shown in Table 2. RCTs with an intervention that consisted of a mobile technology system intended to facilitate weight loss in adults were included in the review. Quasi-experimental and case studies/series were excluded because of the risk of bias associated with these designs 22. Duplicate articles were excluded. Mobile electronic technologies of interest comply with the definition ‘devices which either have interactive wireless cellular communication capability and/or those which run software applications and are highly portable’ 12. Although it was decided that any studies involving large or cumbersome portable technologies should be excluded on the grounds of being ‘left at home’ because of inconvenience, no such studies were found. There is no standard definition of size and weight for handheld or worn technologies so using the wide acceptance of mobile phones as a guide, a pragmatic limitation was chosen. For the purposes of this review mobile technology of interest was refined as follows:

- Portable (smaller than 10 × 15 × 2 cm and weighing less than 200 g) digital technology to run software applications that is carried (in the hand, in a pocket or a bag) or worn (on the body or embedded in clothing) whose interventions can be individually tailored and delivered at any time it is worn or carried in everyday life (through automatic measurements, discrete short message service [SMS], verbal communications or diaries).

**Table 2:** Inclusion/exclusion criteria

Population	Overweight, obese/morbidly obese (body mass index > 25) human adults (age > or = to 18)
Intervention	Mobile technologies excluding only Internet desktop/laptop computers
Comparators	Any other comparator including no treatment/treatment as usual
Outcomes	Primary outcome: weight loss however measured. Secondary outcomes: body mass index, waist circumference, weight maintenance, quality of life/satisfaction with technology
Study design	Randomized controlled trial

Thus, technologies of interest are palmtop computers, mobile phones, PDAs and other handheld, worn or pocket computers.

Two reviewers (RB and CL) independently screened the titles and abstracts of articles retrieved from the database search. Relevant articles were selected according to the inclusion/exclusion criteria, and then two reviewers (RB and CL) agreed on the selected articles as to whether they should be included. Disagreements were resolved by consensus with a third reviewer (PC). The full texts were obtained for in-depth review and data extraction.

### Data extraction

Two independent reviewers used a standardized form to extract data (shown in Table S1, Supporting Information) in the following areas: (1) general information; (2) study characteristics; (3) participant characteristics; (4) intervention and setting; (5) outcome data/results 25. The form was piloted by the two reviewers (RB and CL) before agreeing the content and form of data to be extracted.

### Risk of bias assessment

The risk of bias of the included studies was undertaken independently by two reviewers (RB and CL) using the Cochrane risk of bias tool 26; the guidelines upon which judgements are made are shown in Supporting Information Table S2. A study with a low risk of bias was defined as one fulfilling six or more of the criteria items and with no fatal flaw which is defined as:

1. Dropout > 50%.
2. Statistically and clinically significant differences between groups at baseline indicating unsuccessful randomization.

This approach has previously been validated 27. Any disagreements relating to interpretation of the criteria were resolved through discussion. A third reviewer (PC) was available to arbitrate, but was not needed.

### Data synthesis

Qualitative analysis and comparisons of the articles were conducted (RB and PC). Because of the heterogeneity of the studies retrieved, with regards to the mobile technology systems offered and associated features of interventions, a qualitative synthesis tool using a rating system for levels of evidence 28 was also used (RB and CL). The Van Tulder rating system (shown in Supporting Information Table S3) is used to summarize the results in which the quality and outcomes of individual studies are taken into account.

## Results

### Study selection

**Figure 1:**
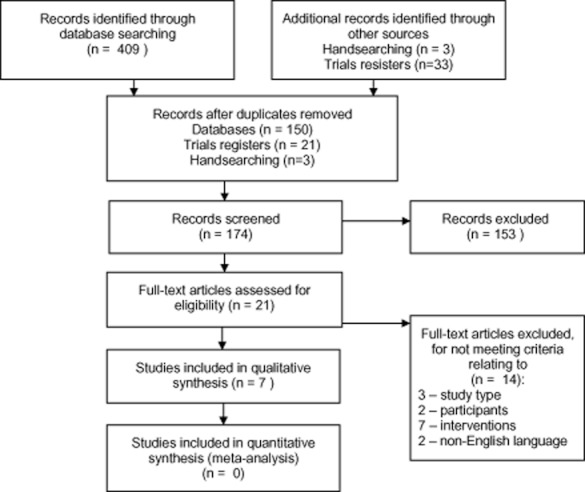
Literature search results.

The review stages are summarized in Fig. 1. In total, 409 articles were identified from the academic databases, three from hand-searching and 33 records were identified from trials registers. These included 65 articles from Medline, 110 from Embase, 104 from Science Citation Index/Social Science Citation Index, 50 from Cumulative Index to Nursing and Allied Health Literature, 80 from The Cochrane Library, 16 from the Metaregister of controlled trials search engine and 17 from Clinicaltrials.gov. Of these, 271 were duplicates, leaving 174 unique articles.

The full text of 21 potentially relevant articles were retrieved and reviewed. Out of these, 14 were excluded for not meeting the criteria relating to study type, participants, interventions and language. A list of the excluded studies can be found in Supporting Information Appendix S1. Data were extracted from the remaining seven academic articles and a structured assessment of the quality of the articles was undertaken in order to review the impact of mobile technology interventions on weight loss.

### The interventions

All the interventions comprised the participants being educated about weight loss via diet and exercise, and carrying with them in their waking hours a mobile device (i.e. a text pager, mobile phone or other) that was a motivator in behaviour change. In some the mobile device was a medium through which the participant received a motivational message. In others, it was used to record what had been eaten, in yet others, it recorded the amount of physical activity, some did both. One technology did all these things by reporting the current daily energy balance. More detail can be found in Tables 4 and 5.

### Risk of bias assessment

The results of the Risk of Bias Assessment are shown in Table 3 where each item was scored as follows: yes (=1), no (=0) and unclear (=0). The level of agreement between reviewers was 85%. Four out of seven studies 29–32 were regarded as presenting a low risk of bias in accordance with the Cochrane guidance 27, and three studies 33–35 were regarded as presenting a high risk of bias.

**Table 3:** Completed risk of bias tool

	Was the method of randomization adequate?	Was the treatment allocation concealed?	Was the patient blinded to the intervention?	Was the care giver blinded to the intervention?	Was the outcome assessor blinded to the intervention?	Was the dropout rate described and acceptable?	Were all randomized participants analysed in the group to which they were allocated?	Free of selective reporting?	Similarity of baseline characteristics?	Co-interventions avoided or similar?	Compliance acceptable?	Timing of outcome assessments similar?	Total
Haapala *et al*. (2009) 30	?	?	?	✓	?	–	✓	✓	✓	–	✓	✓	6
Hurling *et al*. (2007) 31	–	?	–	–	–	✓	✓	✓	✓	?	✓	✓	6
Luley *et al*. (2010) 34	?	?	–	?	–	–	✓	✓	–	?	✓	✓	4
Patrick *et al*. (2009) 35	✓	?	–	–	?	–	✓	✓	–	?	✓	✓	5
Luley *et al*. (2011) 32	✓	?	–	–	–	✓	✓	✓	✓	?	?	✓	6
John *et al*. (2011) 33	?	?	?	?	?	✓	✓	✓	✓	?	?	✓	5
Volpp *et al*. (2008) 29	✓	✓	–	–	–	✓	✓	✓	✓	✓	✓	✓	9

✓, yes; –, no; ?, unclear.

### Study characteristics

A summary of the characteristics of the included studies and main results is provided in Table 4. In some respects, the studies display clear similarities. All the studies in the review 29–35 were community-based and employed mobile technology in an experimental arm that could facilitate weight loss compared with the corresponding control arm. Subjects included in the studies were mixed in terms of gender, but in four out of seven studies there was a significant majority of women (over a 64–80% range). The six studies whose primary focus was weight loss 29,30,32–35 all used independent monitoring of weight and returned feedback to the service user. The other study 31 measured weight objectively before and after the trial. From the five recommended features of technology-based weight loss interventions 10 all the studies could be said to employ a structured programme, which was individually tailored and included self-monitoring and counsellor feedback and communication. None employed follow-ups in excess of one year to examine long-term sustained weight loss (and corresponding behaviour change success).

**Table 4:** Study characteristics and main results

Reference	29	30	31	32	33	34	35
Country	USA	Finland	United Kingdom	Germany	USA	Germany	USA
Experimental group	Text pager Diet and physical activity Self and monthly weigh-ins. + lottery chance OR deposit fined Participants financially incentivized	Mobile phone Diet Self and 3 monthly independent monitoring	Mobile phone and accelerometer Physical activity Self-monitoring and peer support Participants paid	Telemonitoring (weighing scales and accelerometer) Diet and physical activity Self and semi independent daily, weekly feedback, monthly blood samples, monitoring Participants charged	Mobile phone Diet and physical activity Self and monthly weigh-ins. + deposit fined – either 24 or 32 weeks weight loss Participants paid and fined	Telemonitoring (weighing scales and accelerometer) Diet and physical activity Self and weekly independent monitoring; family participation One sub-group received financial incentive	Mobile phone Diet and physical activity Self, weekly and monthly independent monitoring
Comparison	No access to technology, just monthly weigh-ins. Same information as EG	No access to technology and service Less information than EG	Access to accelerometer technology Less information than EG Participants paid	No access to technology and service Less information than EG	No use of mobile phone Same information as EG	No access to technology and service Same information as EG	No access to technology and service Less information than EG
Subjects	N = 57 (F5%, M95%) N = 19 Intervention N = 19 each Control Age 30–70 years (mean = NS) BMI 30–40 kg/m2 (mean = 34.9)	N = 125 (F78%, M22%) N = 62/42 Intervention N = 63/40 Control Age 25–44 years (mean = 38.1) BMI 25–36 kg/m2 (mean = 30.6)	N = 77 (F66%, M34%) N = 47 Intervention N = 30 Control Age 30–55 years (mean = 40.4) BMI 19–30 kg/m2 (mean = 26.3)	N = 70 (F51.5%, M48.5%) N = 35 Intervention N-35 Control Age 48–66 years (mean 57.5 ) BMI 28.9–41 kg/m2 (mean )	N = 66 (F17%, M83%) N = 22 Intervention N = 22 each Control Age 30–70 years (mean = NS) BMI 30–40 kg/m2 (mean = 34.6)	N = 111 adults (F64%, M36%) N = 18 Intervention N = 93 Control Age 20–60 years* (mean = 39*) BMI 26–54* kg/m2 (mean = 33.0*)	N = 78 (F80%, M20%) N = 39 Intervention N = 39 Control Age 25–55 years (mean = 44.9) BMI 25–39.9 kg/m2 (mean = 33.2)
Trial length	16 weeks	52 weeks	9 weeks	26 weeks	24/32 weeks	26 weeks	16 weeks
Targets	0.45 kg per week adjusted at weigh-ins	Chosen by participants	Chosen by participants	Calorific reduction and exercise prescribed	0.45 kg per week adjusted at weigh-ins	Calorific reduction and exercise prescribed	Calorific reduction prescribed; method chosen by participants
Weight loss	EG1 (Deposit contract) 6.3 kg (95% CI, *P* = .006), 10.2 (SD) EG2 (Lottery0 5.9 kg (95% CI, *P* = .02), 12.6 (SD) CG 1.76 kg (mean) (95% CI), 4.1 kg (SD)	EG 5.4% (SD 5.8) 4.5 kg (SD 5.0) CG 1.3% (SD 6.5) 1.1 kg (SD 5.8) Difference 4.1% greater for EG (SD 1.4%) (for a 95% CI quoted as *P* = 0.006)		EG −11.3 plus or minus 5.9 (relative %) −11.8 plus or minus 7.6 (absolute) CG −0.2 plus or minus 2.9% (relative%) −0.3 plus or minus 2.9 (absolute) Difference NS *P* = 0.000 in all cases for weight	EG 3.91 kg, 6.54 (SD) CG 0.53 kg, 6.21 (SD) Difference NS (95% CI, 0.56, 14.50, *P* = .04)	EG 8% CG 4.8% Difference 3.7% greater for EG† (95%CI = 0.9 to 6.5%, *P* = .033)	EG 3.16% CG 1.01% Difference 1.97 kg greater for EG (95% CI = 0.34 to 3.6 kg, *P* = .02)
BMI change			EG −0.24, SE = 0.11 CG +0.10, SE = 0.14 (95% CI = −0.02 to 0.70, *P* = 0.06).	EG −11.3 plus or minus 5.9 (relative %) −4.1 plus or minus 2.6 (absolute) CG −0.2 plus or minus 2.9 (relative %) −0.1 plus or minus 1.0 (absolute)			

This study comprised four different dyads of control and experimental groups where the only difference between control and corresponding experimental group was use of telemedicine.

* Estimates from data supplied about recruits, i.e. not provided explicitly nor as an inclusion/exclusion criteria.

† This value is quoted in the paper but seems is a typo as the difference of published values would be 3.2%.

BMI, body mass index; CG, control group; CI, confidence interval; EG, experimental group; NS, not significant; SD, standard deviation; SE, standard error.

However, in other ways, the studies are different. Five of the RCTs recruited otherwise clinically healthy overweight or obese adults in the USA 29,33, Finland 30, the United Kingdom 31 and Germany 34. The variation of country across the studies indicates the international spread of evidence, but is not in itself a significant factor in the analysis. Another USA study 35 stated that participants were not taking medications known to cause weight gain, and in the other German study 32 participants had elevated levels of plasma glucose and/or HbA1c and/or regular use of anti-diabetic medication. Where stated the mean age of participants ranged from 38 to 58 years and mean body mass index (BMI) also ranged over 26–35 kg/m2. Trial length differed considerably from 9 weeks 31, 16 weeks 29,35, 24/32 weeks 33, 26 weeks 32,34 and 52 weeks 30. Three of the studies 31,32,34 collected continuous automatic activity measurements.

There were financially relevant considerations for participants across the studies, which spanned charging to rewarding participants including doing neither (Table 4). The comparison group in six out of the seven studies 29,30,32–35 had no access to the technology and service whereas one study had a comparator with access to the accelerometer technology, but not the mobile phone 31. The latter study was also different because it was the only one whose primary goal was increased physical activity and not weight loss.

The five recommended key components for effective technology-based weight loss interventions are self-monitoring, counsellor feedback and communication, social support, use of a structured programme, and use of an individually tailored programme 10. Four out of the five recommendations are met by most studies included in our review (30–32,34,35 Table 5), with two studies 29,33 meeting three out of five recommended features 10. However, there are variations in the detail of the components. Additionally, there are many other features that vary across the studies. Table 5 summarizes the five components and many variations. Regarding the self-monitoring feature, four studies monitored weight only 29,30,33,35, one exercise only 31 and two both 32,34. In terms of counsellor feedback and communication, six studies 29–31,33,34 generated automatic feedback to participants, for example by providing counsellor feedback by weekly letter and automatic feedback when logged into the project site 32,34, with the other study 35 adding a monthly phone consultation. Explicitly encouraged opportunity for between study participants social support was not present in most studies 29,30,32–35, but was encouraged in one 31. The degree and form of structuring of the intervention programme varied in many details as can be seen in Table 5. While all the studies employed individually tailored interventions, three studies 32,34,35 specified the aim of reducing the calorific intake while leaving the exact combination of diet and exercise to the individual.

**Table 5:** Study features

Study feature	29	30	31	32	33	34	35
Self-monitoring	Weight	Weight	Exercise	Weight and exercise	Weight	Weight and exercise	Weight
Participants asked to report weight frequently*	✓	✓	X	✓	✓	✓	✓
Technology automatically records physical activity*	X	X	✓	✓	X	✓	X
Counsellor feedback and communication	Daily	No, but auto SMS	No, but auto SMS/e-mail	Weekly by letter	Daily	Weekly by letter, and auto online	Monthly by phone, and auto SMS/mms
Automatic feedback given*	Responsive	Responsive	Responsive	X	Responsive	Responsive	2–5 a day
Human professional feedback given*	X	X	X	weekly	X	weekly	monthly
Social support	No	No	Yes	No	No	Not explicit, but family likely	No, though checked if it was happening
Opportunity for peer support*	X	X	✓	X	X	X	X
Structured programme	✓	✓	✓	✓	✓	✓	✓
Individually tailored	X	✓	✓	✓	X	✓	✓
Goal includes to reduce calorie intake	X	✓	X	✓	X	✓	✓
Weight loss goal set by participants*	X	✓	✓	X	X	X	X
Otherwise explicitly healthy recruits	X	✓	✓	X	X	X	X
Any blinding measures reported	X	✓	X	X	X	X	X
Intervention duration	16 weeks	12 months	9 weeks	6 months	32 weeks	6 months	4 months
All participants receive same advice (re diet and/or exercise)	✓	X	X	X	✓	✓	X
In addition to ‘by interview’ baselines behaviours established over	–	–	3 weeks (activity)	–	–	3 d (diet)	–
Participants record on paper*	X	X	X	X	X	X	✓
Internet key element of intervention*	X	✓	✓	✓†	X	X	✓
Comparator without technology	✓	✓	X	✓	✓	✓	✓
Mid-study independent weight checks*	✓	✓	X	Automatic‡	✓	Automatic‡	✓
Post weight loss follow-up	✓	X	X	X	✓	X	X

* As relevant to experimental group.

† To pass data electronically daily.

‡ Daily measures on bluetooth scales, but could be cheated.

SMS, short message service.

### Weight loss and BMI change results by mobile technology intervention

Weight loss and BMI change results can be observed in Table 4. A reduction in weight of 5–10% is associated with an improvement in the clinical risk of adverse health problems 7. Given that there are significant variations between the interventions in the seven studies reviewed, each study is briefly discussed later.

In one RCT focusing on weight loss following financial incentives and text pager messages 29, there is evidence (n = 57 and 52 completers) with a low risk of bias that mean weight loss at 16 weeks was significantly greater in each of the intervention groups (13.1 lb for the lottery group [*P* = 0.02], 14.0 lb for the deposit contract group [*P* = 0.006] vs. 3.9 lb for the control group). Post-intervention at 7 months, there was still weight loss in the intervention groups, but the difference to the control group was no longer statistically significant.

For a mobile phone-operated weight loss programme vs. no intervention 30, there is evidence from the RCT (n = 125, 82 completers) with a low risk of bias to support additional clinically relevant weight loss in the medium-term (5.4% vs. 1.3% over 12 months, *P* = 0.006). All the participants were free to engage in whatever weight loss activities they wished, and the results are reported to be upheld when these are taken into account.

The Internet- and mobile phone-based physical activity programme study 31 compared ongoing guided/support with use of the technology, and physical activity programme vs. limited advice on exercise and no support with wrist worn accelerometer. The RCT (n = 77 participants and completers) has a low risk of bias and found no significant change in BMI values for the experimental group and control (BMI, −0.24 vs. +0.10, *P* = 0.06). However, the study reports statistically significant lower-percentage body fat in the short-term (−2.18% vs. −0.17% in 9 weeks). It can be seen in Table 4 that the participants had a lower average BMI (26 vs. 30–33 kg m^2^) when compared with the other studies because many participants had normal BMIs and the primary goal was to observe increased activity not weight loss.

In a weight loss RCT with obese patients with type 2 diabetes using telemedical equipment vs. no access to the technology and service 32, there is evidence (n = 70, 68 completers) with a low risk of bias to support weight loss in the intervention group vs. no relevant changes in body weight in the control group over 6 months (11.8 kg ± 8.0 kg compared with 0.3 ± 2.9 kg, *P* = 0.000).

For the weight loss intervention study using financial incentives and mobile phones 33, there is evidence (n = 66 and 60 completers) with a high risk of bias that mean weight loss at 32 weeks was statistically greater in the intervention groups (8.70 lb) relative to the control arm (1.17 lb) (*P* = 0.04). The study did not report percentage reduction in weight or BMI change.

For the telemonitoring intervention with weighing scale and activity monitoring by accelerometer vs. the same training and information without the use of the technology and its services 34, there is evidence from an RCT (n = 142, 111 completers) with a high risk of bias to support clinically relevant weight change in the short-term (6 months). The mean weight loss with telemonitoring was 8.0 vs. 4.8% (*P* = 0.033). The higher percentage is equivalent to an average of 1.25 kg per month for a BMI of around 30. While not clearly stated there is an inference that participants during the study only employed the weight loss interventions they were provided with by the study. However, we note that the physical activity aspect of the intervention is not well described, there is no explanation of what participants in the various groups were told about the role of exercise in their weight loss programme, which may mean they were free to engage or not in this as a co-intervention.

An RCT involving mobile phone feedback message intervention vs. usual care 35 (n = 78, 58 completers) showed evidence with a high risk of bias to support subclinically relevant weight loss in the short-term (3.2% for the experimental group and 1% for the control over 4 months, *P* = 0.02). The usual care comprised the same baseline assessment followed by monthly information or tips about achieving weight loss through managing diet and exercise. For the experimental group, the Internet was used just to choose preferences for tailored messages, otherwise, they used mobile phones to receive and send messages during the self-monitoring. Because the control group is described as ‘usual care’ and the experimental group as usual care plus the study intervention, it is not clear what freedoms the participants had to employ weight loss techniques.

In light of the results mentioned earlier on weight loss across the studies, if the studies are considered to be sufficiently clinically similar, then the level of synthesized evidence according to the Van Tulder evaluation 28 is found to be ‘strong’ as there are consistent findings in multiple high-quality RCTs (>2). However, the evidence is ‘moderate’ in the medium-term (with only one higher quality RCT).

## Discussion

This systematic review summarizes the results of the most robust and well-conducted RCTs available at the time of writing for mobile technology interventions for overweight and obesity 29–35. The trials show consistent evidence that weight loss occurs as a result of mobile technological-based/-assisted interventions, and that clinically significant weight loss is achievable for at least a proportion of overweight or obese participants.

### Weight loss

From the available evidence, one study 31 aimed at increasing exercise did not report weight loss, nor was there statistically significant BMI change. Another study 35 did not meet the 5–10% clinical standard of weight loss 7. Three studies found greater weight loss in the intervention groups 29,32,33, but this was reported in pounds 29,33 and kilogrammes 32 rather than percent body weight loss. Two studies did meet the recommended clinical standard of 5–10% weight loss 30,34 but only one of these studies 30 had a low risk of bias 26. Therefore, although weight loss occurs with the use of mobile technology, the results of the available evidence should be treated with caution (Table 4).

It is worth pointing out that none of the studies (apart from 33 that followed-up after 36 weeks) conducted a post-intervention follow-up to determine sustained long-term benefits or behaviour change, and none of the studies show long-term weight loss. Six of the interventions are of 6 months duration or significantly less, and just one study 30 utilized a longer intervention period. In the United Kingdom, for example, the National Obesity Observatory 36 recommends a minimum of three follow-up points, including at one year. Follow-up data on key measures such as height, weight, physical activity and diet should be over a greater term than one year. In line with the previous source 36 the latter is employed for the definition of long-term follow-up.

In one study 30 the rate of loss of weight is maximized during the first 3 months but a peak in net weight loss occurs at 6 months and then regression is seen. Nonetheless about 25% of subjects lose a clinically significant amount of weight at 12 months and the study has a low risk of bias. Two other studies with a low risk of bias report significantly greater weight loss in the intervention group 29,32, but these only cover 16 weeks and 6 months duration, respectively. The final study with a low risk of bias 31 reports no significant change in BMI and lower-percentage body fat, but this only covers a 9-week period. Therefore, the shorter duration studies could be viewed as designs that will overestimate the longer-term benefit – with three of them operating over an approximate period of the most rapid weight change 29,31,35 and three finishing at a point where maximal weight change from an intervention may be seen 32–34.

The results of this review are that three out of four individual low risk of bias studies each support the use of mobile interventions and, if considered sufficiently similar, according to the measure of quality 28 present strong evidence that mobile interventions lead to successful weight loss in overweight and obese adults with a BMI of 25–39.9. This finding agrees with the inferred success of wider technological-based interventions 10,37.

### Recommendations for improving the quality and reporting of future trials

Despite the stated strong evidence 28, shortcomings in the evidence have been identified. Not surprisingly, this suggests further research is warranted to better determine long-term benefits and tackle the restricting factors described later.

The generalizability of some of the included studies may be limited because of the small sample size 38, relatively narrow age range of users and length of study 29,31,35. For example, the duration of included trials varied considerably from 9 weeks to 12 months and future research should be of sufficient power, duration and long-term follow-up to ensure detection of behaviour change. Another recommendation is that subjects could include ‘healthy’ adults, but also those with one or more chronic conditions as this latter group are only covered by one 32 of the seven RCTs contained in this review.

The risk of bias assessment (Table 3) highlights that three out of the seven studies 33–35 presented a high risk of bias. The results of the reviewed studies should be interpreted with caution and further research should consider the guidelines for assessing risk of bias (shown in Supporting Information Table S2), such as the method of randomization and the use of blinding 26 to ensure high-quality study design.

Apart from the care giver blinding in one study 30 a consistent feature across all included studies is a failure to conceal treatment allocation, and blinding of patient, care givers and outcome assessor to the intervention (Table 3). These shortcomings are widely regarded as typical in pragmatic studies of this nature 39. However, it is important to recognize the possible influence of care giver and patient expectations or preferences upon treatment outcome in terms of an under or over exaggeration of treatment effect 40. While, *a priori*, participants cannot be blinded to their own behaviour change intervention that they actively manage themselves, it is possible to blind the participants and others, including outcome assessors, to which arm of the trial they are in and often confounding details of the aims of the study 41. Therefore, adoption of blinding techniques will improve the quality of evidence.

Cost-effectiveness of the interventions was not addressed by any of the studies included in the review and should be included in future research given the high cost associated with overweight and obesity 3,6. Future research should verify the utility of the five key components for successful weight loss interventions 10 such as self-monitoring of weight, exercise and diet, all of which in principle may improve effectiveness (Table 5).

It is well accepted and published that incentives such as the payment of participants can be a successful intervention for weight loss 29,33, but it is difficult to ascertain the effect of financial incentive in the other three studies where this was an issue 31,32,34. In one study 31, both the intervention and experimental arms received payment so while this may have influenced the motivation to participate, it is not possible to identify how this influenced weight loss as there is no comparator. In a second study 34, one subgroup in the experimental arm received a financial incentive and had greater weight loss indicating that this is a viable strategy, whereas in another study 32, body weight was lowered when participants were charged to take part. Future research could consider including financial incentives.

To ensure transfer of any new effective intervention programmes from research study to implemented care, it is essential to recognize this requires changing the ways existing services work. Central to achieving effective transfer is consideration of and with the individuals involved 42, in this case those seeking to lose weight, obesity practitioners and commissioners of services. Early involvement of representatives of such stakeholders in research and development studies is warranted.

### Limitations of the included studies

Because of the high risk of bias of three of the studies 33–35, and the follow-up lengths of all included studies, the results should be treated with caution. The concentration on ‘otherwise healthy subjects’ (apart from 32) misses out those populations with chronic conditions associated with obesity such as diabetes and stroke. Also, the participants may have potentially come from a certain level of literacy and socioeconomic status because of the use of mobile phone and Internet resources within the interventions. Three studies 29,32,33 do not report percentage weight loss and one study 31 fails to report a percentage change in BMI or weight loss. This makes it difficult to readily compare with the recommended clinical standard of 5–10%.

It appears the included studies did not use a systematic mixed methods approach 43 and future research could consider this as well as guidelines for designing complex interventions 44. A useful metric for practitioners of the impact on populations would seem to be the percentage of participants who obtained clinically useful weight loss; this was reported only in one study. None of the studies distinguished weight loss in two separate stages of initial weight loss followed by maintenance. Future research could measure the success of mobile interventions in the different stages.

### Strengths and limitations of this review

The strengths of this systematic review relate to the methodology employed 22, and the unique assessment of risk of bias 26 compared with other reviews in the area. It establishes the strong level of evidence for mobile-technology interventions for obesity in the short-term and moderate evidence in the medium-term. Recommendations for future study design and longer-term studies are made, and the definition of mobile technology may have also added to the field. However, it should be noted that the practical boundaries concerning portability are constantly changing. For example, Internet access is now widely accessible on reasonably portable devices.

Limitations include the fact that terms could have been missed in the search strategy if they were not mentioned in the title/abstract. Finally, our definition of technology was mobile and therefore excluded technology like the Internet, but these technologies have been reviewed elsewhere 10.

It is difficult to classify, compare and contrast the features of the studies and interventions because of the heterogeneous nature of the studies reviewed (Tables 4 and 5), and because of incomplete/unclear reporting. The former is a reflection of a number of factors such as the large potential for variation of intervention protocol, studies in different countries where the ‘standard’ care given for obesity differs, and the relatively few RCT studies reported in the area. However in a recent meta-analysis of 22 studies employing mobile interventions for diabetes mellitus, where diet and exercise control was involved in 50% of them, it was found that many variations in the details did not significantly impact on the outcomes 37. Nonetheless, the complexity of the services around the technologies themselves means it is difficult to discriminate the key factors in the interventions that drove the weight loss. Given the variation in the studies, future research should limit the differences in variables such as study design, subjects enrolled, interventions applied and devices employed, and assess the impact of the different variables.

## Summary of recommendations for future RCT studies of mobile-based interventions

Future trials should have the following features to ensure high-quality design. Research should: involve stakeholders 42; take a mixed-methods approach 43; take steps to reduce bias, e.g. employ blinding where possible 26 and avoid confounding interventions; and follow guidelines for assessing the risk of bias 26. When employing complex interventions 44, they should be of sufficient duration and have long-term follow-up 36 and consider the five key components for successful weight loss 10. Cost-effectiveness of interventions should be assessed and inclusion of financial incentives considered. Reporting should: adhere to CONSORT guidance 45, describe the inclusion or not of the five key components for successful weight loss 10, report weight loss or BMI results in percentage terms to enable meta-analyses and the clinical standard of 5–10% weight loss to be assessed 7, and to report the percentage of participants in the recruited study arms who achieved clinically useful weight loss.

## Conclusions

If the trials included are considered sufficiently clinically similar, as might be inferred from the meta-analysis by Liang 37, then it can be stated according to Van Tulder 28 there is strong research evidence for short-term and moderate evidence for medium-term weight loss through use of mobile technologies as part of intervention delivery. However, the required assumption of clinical similarity and limitations of the studies as reported suggest further research is still needed. Future research needs to involve well-designed high-quality pilot and subsequent scaled up RCTs that report results that facilitate meta-analyses, evaluation of the long-term benefit and cost-effectiveness of interventions employing mobile technology. While there is a recommendation on key features to include in weight loss interventions using information technology 10 it is not based on strong evidence. Thus, research could also be planned to better understand (or even determine) the optimum arrangement of the many details of interventions using mobile technologies. However, stopping the former research to first investigate the latter would delay the potential benefits reaching patients. A balanced approach would therefore be for both types of study to be pursued.

## Supporting information

**Table S1.** Data extraction form.

**Table S2.** The Cochrane Back Review Group guidelines for assessing risk of bias (Furlan *et al*. 26).

**Table S3.** Levels of Evidence (van Tulder *et al*. 28).

**Appendix S1.** List of excluded studies.
