# Supplementary material for: Interventions employing mobile technology for overweight and obesity: an early systematic review of randomized controlled trials
Source: Obes Rev. 2012 Nov 20;14(4):279–91. doi: 10.1111/obr.12006 (PMC3618375; doi:10.1111/obr.12006)
Supplement: Supplementary file 1 [file obr0014-0279-SD1.docx]

**Supporting Information**

Interventions employing mobile technology for overweight and obesity: an early systematic review of randomised controlled trials.

Authors: Ruth Bacigalupo, Peter Cudd, Chris Littlewood, Paul Bissell, Mark S Hawley and Helen Buckley Woods

Affiliation: School of Health and Related Research (ScHARR), University of Sheffield, UK

Address for correspondence:

Ruth Bacigalupo

School of Health and Related Research

University of Sheffield

Regent Court

30 Regent Street

Sheffield

S1 4DA

[R.Bacigalupo@sheffield.ac.uk](mailto:R.Bacigalupo@sheffield.ac.uk)

**Table S1, Data Extraction Form**

| **General information**  Researcher performing data extraction:    Date of data extraction:    Identification features of the study:  Author/ Article title:  Type of publication (e.g. journal article, conference abstract):  Country of origin (language): |
| --- |
| **Study characteristics**  Aim/objectives of the study:  Study design:  Study inclusion and exclusion criteria  Recruitment procedures used (e.g. details of randomisation, blinding) |
| **Participant characteristics**  Characteristics of participants at the beginning of the study e.g. Age/ Gender/ Ethnicity/ Socio-economic status/ Disease characteristics/ Co-morbidities  Number of participants in each characteristic category for intervention and control group(s) or mean/median characteristic values (record whether it is the number eligible, enrolled, or randomised that is reported in the study) |
| **Intervention and setting**  Setting in which the intervention is delivered:    Description of the intervention(s) and control(s) (e.g. dose, number of cycles, duration of cycle, care provider):    Description of co-interventions: |
| **Outcome data/results**  Unit of assessment/analysis    Statistical techniques used:    For each pre-specified outcome:  Whether reported:  Definition used in study:  Measurement tool or method used:  Length of follow-up, number and/or times of follow-up measurements    For all intervention group(s) and control group(s):  Number of participants enrolled  Number of participants included in analysis  Number of withdrawals, exclusions, lost to follow-up  Summary outcome data e.g.  Dichotomous: number of events, number of participants  Continuous: mean and standard deviation    Type of analysis used in study (e.g. intention to treat, per protocol)  Results of study analysis e.g.  Dichotomous: odds ratio, risk ratio and confidence intervals, p-value  Continuous: mean difference, confidence intervals    If subgroup analysis is planned the above information on outcome data or results will need to be extracted for each patient subgroup    Additional outcomes    Record details of any additional relevant outcomes reported |

**Table S2, The Cochrane Back Review Group Guidelines for Assessing Risk of Bias (Furlan et al. 2009)**

| 1 | **Was the method of randomisation adequate?**  A random (unpredictable) assignment sequence. Examples of adequate methods are coin toss (for studies with 2 groups), rolling a dice (for studies with 2 or more groups), drawing of balls of different colours, drawing of ballots with the study group labels from a dark bag, computer-generated random sequence, pre-ordered sealed envelopes, sequentially-ordered vials, telephone call to a central office, and pre-ordered list of treatment assignments Examples of inadequate methods are: alternation, birth date, social insurance/ security number, date in which they are invited to participate in the study, and hospital registration number. |
| --- | --- |
| 2 | **Was the treatment allocation concealed?**  Assignment generated by an independent person not responsible for determining the eligibility of the patients. This person has no information about the persons included in the trial and has no influence on the assignment sequence or on the decision about eligibility of the patient. |
| 3 | **Was the patient blinded to the intervention?**  This item should be scored “yes” if the index and control groups are indistinguishable for the patients or if the success of blinding was tested among the patients and it was successful. |
| 4 | **Was the care giver blinded to the intervention?**  This item should be scored “yes” if the index and control groups are indistinguishable for the care providers or if the success of blinding was tested among the care providers and it was successful. |
| 5 | **Was the outcome assessor blinded to the intervention?**  Adequacy of blinding should be assessed for the primary outcomes. This item should be scored “yes” if the success of blinding was tested among the outcome assessors and it was successful or:   - for patient-reported outcomes in which the patient is the outcome assessor (*e.g.*, pain, disability): the blinding procedure is adequate for outcome assessors if participant blinding is scored “yes” - for outcome criteria assessed during scheduled visit and that supposes a contact between participants and outcome assessors (*e.g.*, clinical examination): the blinding procedure is adequate if patients are blinded, and the treatment or adverse effects of the treatment cannot be noticed during clinical examination - for outcome criteria that do not suppose a contact with participants (*e.g.*, radiography, magnetic resonance imaging): the blinding procedure is adequate if the treatment or adverse effects of the treatment cannot be noticed when assessing the main outcome - for outcome criteria that are clinical or therapeutic events that will be determined by the interaction between patients and care providers (*e.g.*, co-interventions, hospitalization length, treatment failure), in which the care provider is the outcome assessor: the blinding procedure is adequate for outcome assessors if item “4” (caregivers) is scored “yes” - for outcome criteria that are assessed from data of the medical forms: the blinding procedure is adequate if the treatment or adverse effects of the treatment cannot be noticed on the extracted data |
| 6 | **Was the drop-out rate described and acceptable?**  The number of participants who were included in the study but did not complete the observation period or were not included in the analysis must be described and reasons given. If the percentage of withdrawals and drop-outs does not exceed 20% for short-term follow-up and 30% for long-term follow-up and does not lead to substantial bias a “yes” is scored. (N.B. these percentages are arbitrary, not supported by literature). |
| 7 | **Were all randomised participants analysed in the group to which they were allocated?**  All randomised patients are reported/analyzed in the group they were allocated to by randomization for the most important moments of effect measurement (minus missing values) irrespective of non-compliance and co-interventions. |
| 8 | **Are reports of the study free of suggestion of selective outcome reporting?**  In order to receive a “yes”, the review author determines if all the results from all pre-specified outcomes have been adequately reported in the published report of the trial. This information is either obtained by comparing the protocol and the report, or in the absence of the protocol, assessing that the published report includes enough information to make this judgment. |
| 9 | **Were the groups similar at baseline regarding the most important prognostic indicators?**  In order to receive a “yes”, groups have to be similar at baseline regarding demographic factors, duration and severity of complaints, and value of main outcome measure(s). |
| 10 | **Were co-interventions avoided or similar?**  This item should be scored “yes” if there were no co-interventions or they were similar between the index and control groups. |
| 11 | **Was the compliance acceptable in all groups?**  The reviewer determines if the compliance with the interventions is acceptable, based on the reported intensity, duration, number and frequency of sessions for both the index intervention and control intervention(s). For example, physiotherapy treatment is usually administered over several sessions; therefore it is necessary to assess how many sessions each patient attended. For single session interventions (e.g., surgery), this item is irrelevant. |
| 12 | **Was the timing of outcome assessment similar in all groups?**  Timing of outcome assessment should be identical for all intervention groups and for all important outcome assessments. |

**Table S3, Levels of Evidence (van Tulder et al. 2003)**

| Strong Evidence | Consistent findings in multiple high quality RCTs (n> 2) |
| --- | --- |
| Moderate Evidence | Consistent findings among multiple lower quality RCTs and/ or 1 higher quality RCT |
| Limited Evidence | Only one relevant low quality RCT |
| Conflicting evidence | Inconsistent findings amongst multiple RCTs |
| No evidence from trials | No RCTs |

**Table S4, List of Excluded Studies**

Burke, L. E., Styn, M. A., Glanz, K. et al., SMART trial: a randomised clinical trial of self –monitoring in behavioural weight management-design and baseline findings. *Contemporary Clinical Trials.* 2009; **30**(6): 540-51.

Goulis, D. G., Giaglis, G. D., Boren, S. A. et al., Effectiveness of home-centred care through telemedicine applications for overweight and obese patients: a randomised controlled trial. *International Journal of Obesity and Related Metabolic Disorders: Journal of the International Association for the Study of Obesity.* 2004; **28**(11): 1391-98.

Izquierdo, R., Lagua, C. T., Meyer, S. et al., Telemedicine intervention effects on waist circumference and body mass index in the IDEATel project. *Diabetes Technology and Therapeutics.* 2010; **12**(3): 213-20.

Kortke, H., Frisch, S., Zittermann, A. et al. A telemetrically-guided program for weight reduction in overweight subjects (the SMART study). [German]. *Deutsche Medizinische Wochenschrift.* 2008; **133**(24): 1297-303.

Mattila, E., Parkka, J., Hermersdorf, M. et al. Mobile diary for wellness management – results on usage and usability in two user studies. *Ieee Transactions on Information Technology in Biomedicine.* 2008; **12**(4): 501-12.

McDoniel, S. O., Wolskee, P., and Shen, J. Treating obesity with a novel hand-held device, computer software program, and Internet technology in primary care: the SMART motivational trial. *Patient Education and Counseling.* 2010; **79**: 185-91.

Rossi, M. C. E., Nicolucci, A., Bartolo, P. D. et al. A new telemedicine system enabling flexible diet and insulin therapy while improving quality of life: an open-label, international, multicenter, randomised study. *Diabetes Care.* 2010; **33**(1): 109-15.

Rossi, M. C. P., Perozzi, C., Consorti, C. et al. (2010). An interactive diary for diet management and a new telemedicine system able to promote body weight reduction, nutritional education, and consumption of fresh local products. *Giornale Italiano di Diabetologia e Metabolismo.* 2010; **30**(1): 4-12.

Spring, B., Janke, A., Duncan, J. M. et al. Randomised controlled trial of a personal digital assistant (PDA) as adjunct to group obesity treatment for veterans. *Obesity.* 2010; **18**(S83).

Stone, R. A., Rao, R. H., Sevick, M. A. et al. . Active care management supported by home telemonitoring in veterans with type 2 diabetes the DiaTel randomised controlled trial. *Diabetes Care.* 2010; **33**(3): 478-84.

Tanaka, M., Adachi, Y., Adachi, K., and Sato, C. (2010). Effects of a non-face-to-face behavioural weight-control program among Japanese overweight males: a randomised controlled trial. *International Journal of Behavioural Medicine.* 2010; **17**(1): 17-24.

Thomas, D., Vydelingum, V., and Lawrence, J. E-mail contact as an effective strategy in the maintenance of weight loss in adults. *Journal of Human Nutrition and Dietetics.* 2011; **24**(1): 32-38.

VanWormer, J. J., Martinez, A. M., Benson, G. A. et al. Telephone counselling and home telemonitoring: the weigh by day trial. *American Journal of Health Behaviour.* 2009; **33**(4): 445-54.

Weinstock, R. S. B. Lessened decline in physical activity and impairment of older adults with diabetes with telemedicine and pedometer use: results from the IDEATel study. *Age and Ageing.* 2011; **40**(1): 98-105.
